# Comprehensive Analysis of Cuproptosis-Related Genes According to Cancer Stage and Their Prognostic Value in Cervical Cancer

**DOI:** 10.3390/ijms27177730

**Published:** 2026-08-28

**Authors:** Jing He, Yueyan Sun, Zhonghua Yang, Duo Xu, Pengxia Zhang, Jiaqi Xia

**Affiliations:** 1 School of Basic Medical Sciences, Jiamusi University, Jiamusi 154007, China; 2 School of Clinical Medical Sciences, Jiamusi University, Jiamusi 154007, China

**Keywords:** cervical cancer, cuproptosis-related genes, prognostic model, cancer staging model, machine learning algorithm

## Abstract

Cuproptosis is a novel form of metabolism-associated cell death. Cervical cancer (CC) exhibits elevated serum copper levels and mitochondrial metabolic reprogramming, making cuproptosis-related genes (CRGs) potentially critical for prognosis prediction and therapeutic targeting. However, studies on CRGs in CC remain limited. This study aimed to construct prognostic and cancer staging models for CC using machine learning (ML) algorithms. Gene expression profiles of patients with CC were obtained from the TCGA and GEO databases. Five ML algorithms were employed to identify significant factors, including random forest (RF), support vector machine (SVM), Gaussian mixture model (GMM), Bayesian, and StepCox. A prognostic model was subsequently constructed using LASSO–Cox regression based on the selected genes. Concurrently, a cancer staging model was built using ML algorithms incorporating three distinct gene categories. Finally, qRT-PCR and Western blotting were conducted to validate the expression of signature genes at both the tissue and cellular levels. Additionally, CTD-based screening and in vitro functional assays were performed to evaluate the effects of DDP on CC cells. Through integrated bioinformatics and ML approaches, a prognostic model comprising nine CRGs was successfully established (GMM = 0.72). The derived risk score served as an independent prognostic indicator for CC (*p* < 0.001, 95% CI: 3.681 [1.785–7.591]). Calibration curves confirmed that the nomogram accurately predicted overall survival (OS) at 1, 3, and 5 years. Additionally, a cancer staging model was effectively constructed using the GMM algorithm (AUC = 0.74). DDP dose-dependently inhibited CC proliferation/migration and down-regulated CRG expression. In this study, we developed two different models—a cuproptosis-related prognostic model and a cancer staging model—that highlight promising biomarkers for predicting patient prognosis and cancer progression in patients with CC.

## 1. Introduction

Cervical cancer (CC) is a prevalent gynecological malignancy and ranks as the third most common cancer among women worldwide, following breast and colorectal cancers [1], with 604,127 new cases of CC and 341,831 CC-related deaths reported worldwide in 2020 [2]. CC is primarily composed of squamous cell carcinoma and adenocarcinoma [3] and is a leading cause of death among women in developing countries [4], likely because of reduced access to screening and inadequate preventive measures [1]. Screening consists of performing Papanicolaou tests (Pap test) or human papillomavirus (HPV) tests [5]. Although the development of vaccines against HPV of different genotypes has helped to reduce HPV-related morbidity and mortality, the disease remains widespread and poses a serious threat to women’s health [6,7]. A comprehensive prevention strategy should integrate screening, vaccination, health promotion, and active risk-factor management to achieve synergistic protective effects. The Simple Hysterectomy and Pelvic Node Assessment (SHAPE) trial demonstrated that for low-risk patients with CC, with lesions ≤ 2 cm and limited stromal invasion, simple hysterectomy yields survival outcomes comparable to radical hysterectomy while being associated with fewer complications [8,9]. Currently, surgical intervention represents the therapeutic standard of care; however, adjuvant therapies may improve prognosis in selected cases. For patients at high risk of recurrence, concurrent chemoradiotherapy may be warranted [10]. Among chemotherapeutic agents, cisplatin (DDP) remains the cornerstone of first-line systemic therapy for advanced and recurrent CC, yet response rates are limited by acquired resistance and dose-dependent toxicities. Identifying the molecular determinants of DDP sensitivity could, therefore, refine patient selection and improve therapeutic outcomes. To our knowledge, similar applications of machine learning (ML) algorithms for the prediction of survival status in patients with CC have rarely been reported in the literature. Therefore, further research is needed to identify new prognostic biomarkers to improve treatment and prognosis.

Cancer staging is a fundamental tool for determining the extent of cancer dissemination within the body, thereby informing disease severity and guiding optimal therapeutic approaches. Furthermore, it is utilized by medical professionals in the calculation of survival statistics [11]. According to the International Federation of Gynecology and Obstetrics (FIGO) 2018 revised staging, CC has four distinct stages—stages I, II, III, and IV [12]. Cancer staging provides critical information regarding tumor location, size, and extent of invasion into adjacent tissues. Tumor size has been previously demonstrated to significantly impact overall survival (OS) and represents an important independent prognostic factor associated with increased recurrence risk [13]. Here, the results of the constructed cancer staging model will help us achieve better outcomes for CC in early and late diagnosis. In CC, the early clarification of staging and early treatment are conducive to improving patient survival.

Copper, a transition metal and a vital trace element, plays an essential role in every living organism [14]. However, excessive intake beyond the safe dose is extremely toxic to cells [15]. As copper is necessary for cancer growth and metastasis [16], in vivo studies of it have generated a great deal of attention and have become an important research focus in tumor treatment [17]. In March 2022, a study published in *Science* proposed a new mechanism for copper-induced cell death [18]. Cuproptosis is regulated by a fundamental cellular mechanism involving protein lipoylation. Key players in this process, such as FDX1 [19] and the lipoic acid–related genes LIAS and LIPT1, have emerged as crucial regulators of cuproptosis and are recognized as significant markers of this biological phenomenon [20]. It is noteworthy that CDKN2A has been linked to cellular susceptibility to cuproptosis [21]. One study has found conflicting associations between the aberrant expression of known tumor suppressor genes—including CDKN2A, which codes for the p16INK4A and p14ARF proteins—and CC [22]. Cuproptosis demonstrates a high correlation with cellular metabolism, and certain cancers characteristically exhibit elevated aerobic respiration rates [23]. Moreover, serum copper levels are raised among patients with CC, and changes in copper homeostasis appear critical for CC progression [24]. Since there are few reports on these relationships, we aimed to integrate them to explore the value of cuproptosis-related genes (CRGs) in predicting prognosis and response to immunotherapy in patients with CC.

ML, a branch of artificial intelligence (AI), is increasingly used for disease diagnosis, prognosis prediction, recurrence risk assessment, and complication management [25]. ML algorithms such as random forest (RF), support vector machine (SVM), and logistic regression (LR) have been studied for use in constructing early CC metastasis prediction models [26,27]. SVM learning is a potent classifier that is used for the genomic classification of cancer or categorizing them into subtypes [28]. Moler et al. [29] applied SVM in the classification of colon cancer tissue using selected features. Therefore, using ML models to integrate and analyze these large and complex datasets has become increasingly important to assist physicians’ decision-making.

**Figure 1:**
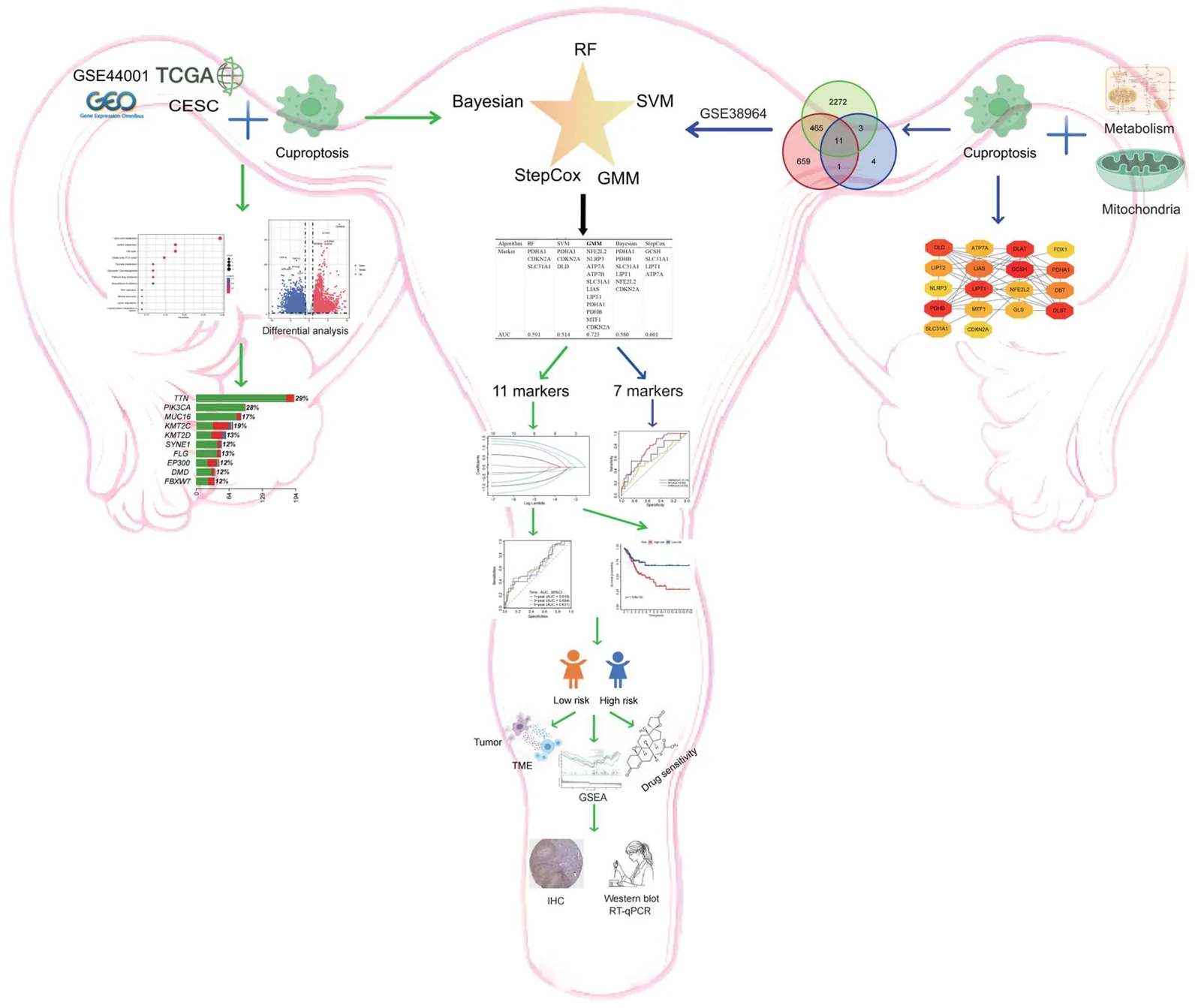
Schematic outline of the study. Green arrows represent the prognostic model, and blue arrows represent the cancer staging model.

This study employed integrated bioinformatics approaches and ML algorithms to construct prognostic risk scores and cancer staging models. Subsequent analysis revealed that CRGs may be valuable for prognosis evaluation and immunotherapy selection. In addition, we verified the expression of CDKN2A, PDHA1, and NFE2L2 in cells and tissues, which might be a promising therapeutic target for the prognosis of CC. The entire analysis workflow is illustrated in Figure 1. The research aims to contribute to the evolving landscape of CC, offering new avenues for therapeutic intervention.

## 2. Results

### 2.1. Landscape of CRGs in CC

**Figure 2:**
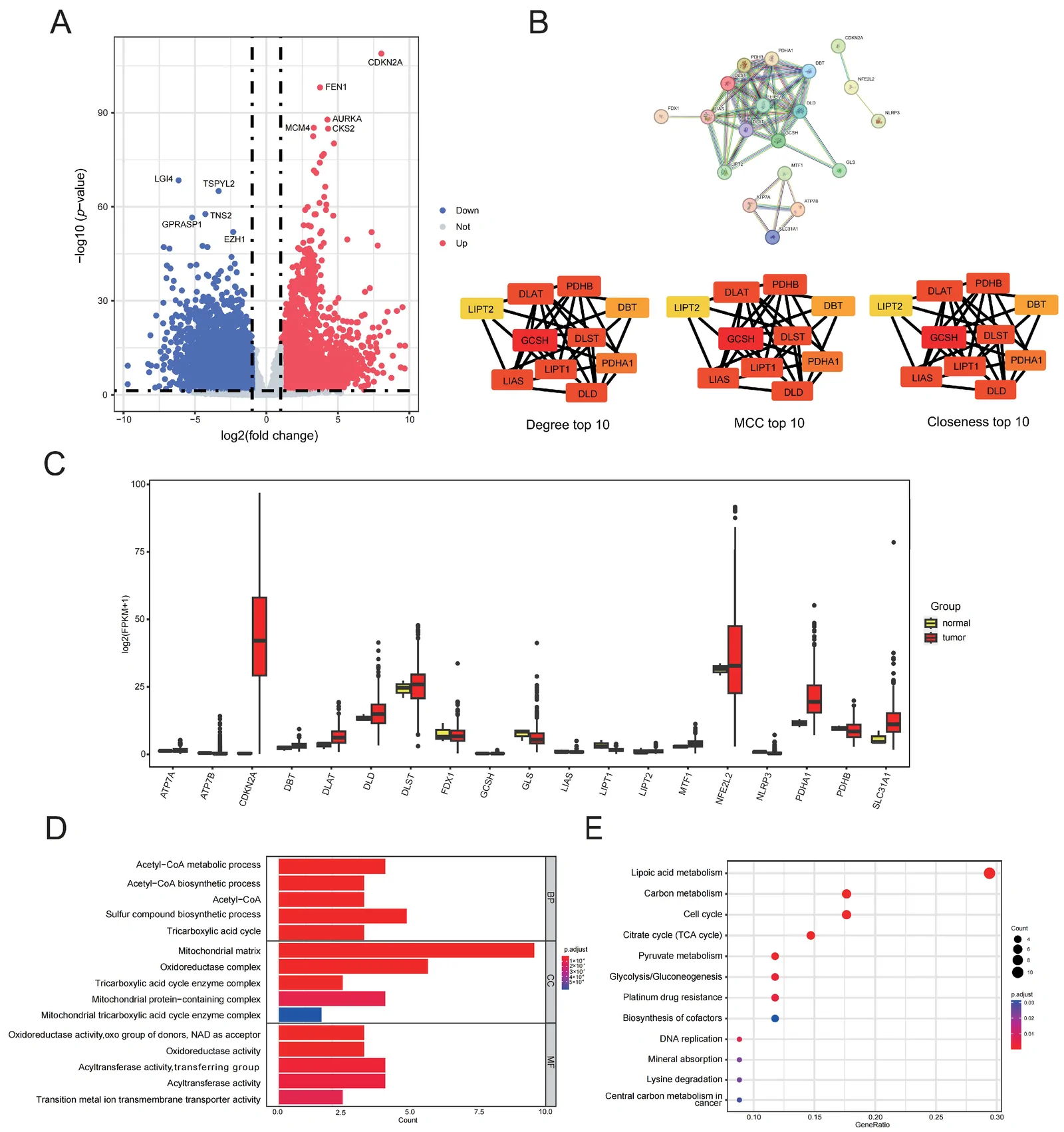
Characterization of CRGs and DEGs in CC: (**A**) differentially expressed genes between normal and CC tissues in TCGA data; (**B**) the protein–protein interaction (PPI) network shows the interactions of CRGs; (**C**) GO enrichment analysis of CRGs and DEGs (BP: biological process, CC: cell component, and MF: molecular function); (**D**) analysis of CRGs and DEGs for KEGG; (**E**) the distinct expression of CRGs between normal and tumor tissues, with red boxes representing tumor groups and yellow boxes representing normal groups.

**Figure A1:**
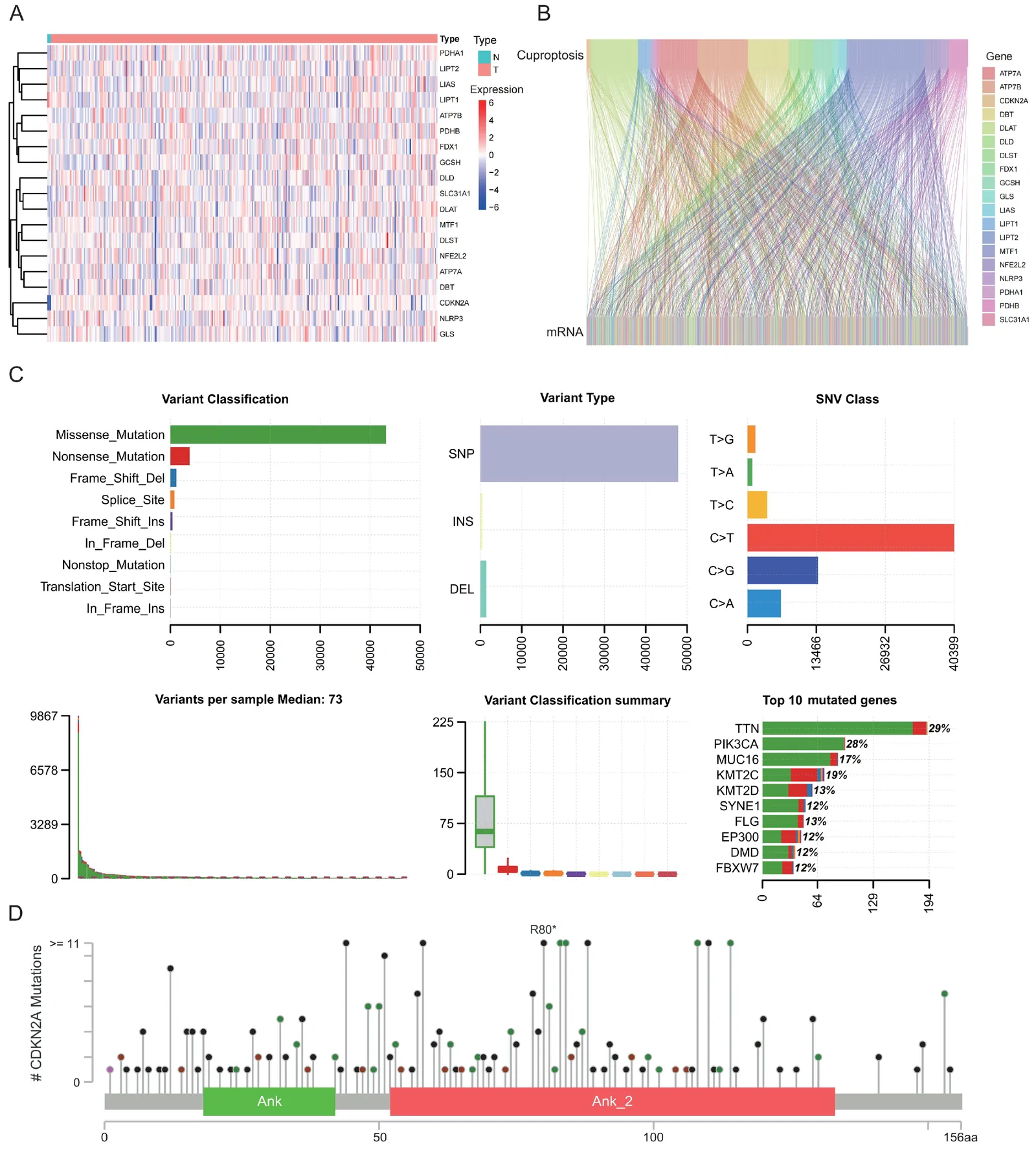
Transcriptional and genetic alterations of DEGs in CC: (**A**) heatmap of 19 CRGs between normal and tumor tissue groups; (**B**) Sankey diagram depicting the mRNAs associated with CRGs; (**C**) mutation frequencies of DEGs in CC from the TCGA cohort; (**D**) mutation frequency of CDKN2A in cervical cancer.

Data were obtained from the TCGA and GEO databases, and prognostic and cancer staging models were constructed and validated using ML algorithms. Differential expression analysis between normal and tumor tissues was performed, followed by functional enrichment and TME analysis across distinct risk groups. Key genes were subsequently validated via Western blot and qRT-PCR analyses. Based on the cuproptosis mechanism described in prior studies, a total of 19 CRGs were selected for further analysis. A total of 9420 differentially expressed genes were identified between normal and CC cells (*p* < 0.05, |logFC| > 1), including 4728 up-regulated genes and 4692 down-regulated genes (Figure 2A). Among the differentially expressed genes, CRG CDKN2A exhibited the most pronounced up-regulation (logFC = 8.04, *p* = 1.24 × 10^−9^). To show associations between the 19 CRGs, a protein–protein interaction network was mapped with the STRING database. Through the analysis methods of degree, MCC, and closeness, we further confirmed their interaction (Figure 2B). We performed data visualization of the expression levels of 19 CRGs between CC and normal tissue samples (Figure 2C and Figure A1A). The results indicate that six CRGs (CDKN2A, DLD, DLAT, PDHA1, SLC31A1, and NFE2L2) were more highly expressed in tumor tissue, whereas LIPT1 showed higher expression in normal tissues compared to tumor tissues. A Sankey diagram was generated to demonstrate the relationship between mRNAs and CRGs in patients with CC (Figure A1B). To characterize mutations affecting differentially expressed genes in CC, the frequencies of copy number variations and somatic mutations were quantified. The top ten genes by mutation frequency are presented in Figure A1C, with CDKN2A mutations identified in 0.5% of the 10,000 analyzed clinical cases (Figure A1D). The analysis of GO enrichment revealed that these genes are enriched in biological processes, such as the metabolic process of acetyl-Coenzyme A, the biosynthesis process of acetyl-Coenzyme A, and the tricarboxylic acid cycle; cellular components, including the mitochondrial matrix, oxidoreductase complex, and TCA cycle enzyme complex; and molecular functions, such as oxidoreductase activity and acyl group transfer (Figure 2D). The KEGG analysis shows that these differentially expressed genes are significantly enriched in pathways such as lipoic acid metabolism and the tricarboxylic acid cycle (Figure 2E). This indicates that CRGs influence CC initiation and progression.

### 2.2. Consensus Clustering Identified Two Clusters of Patients with CC

**Figure A2:**
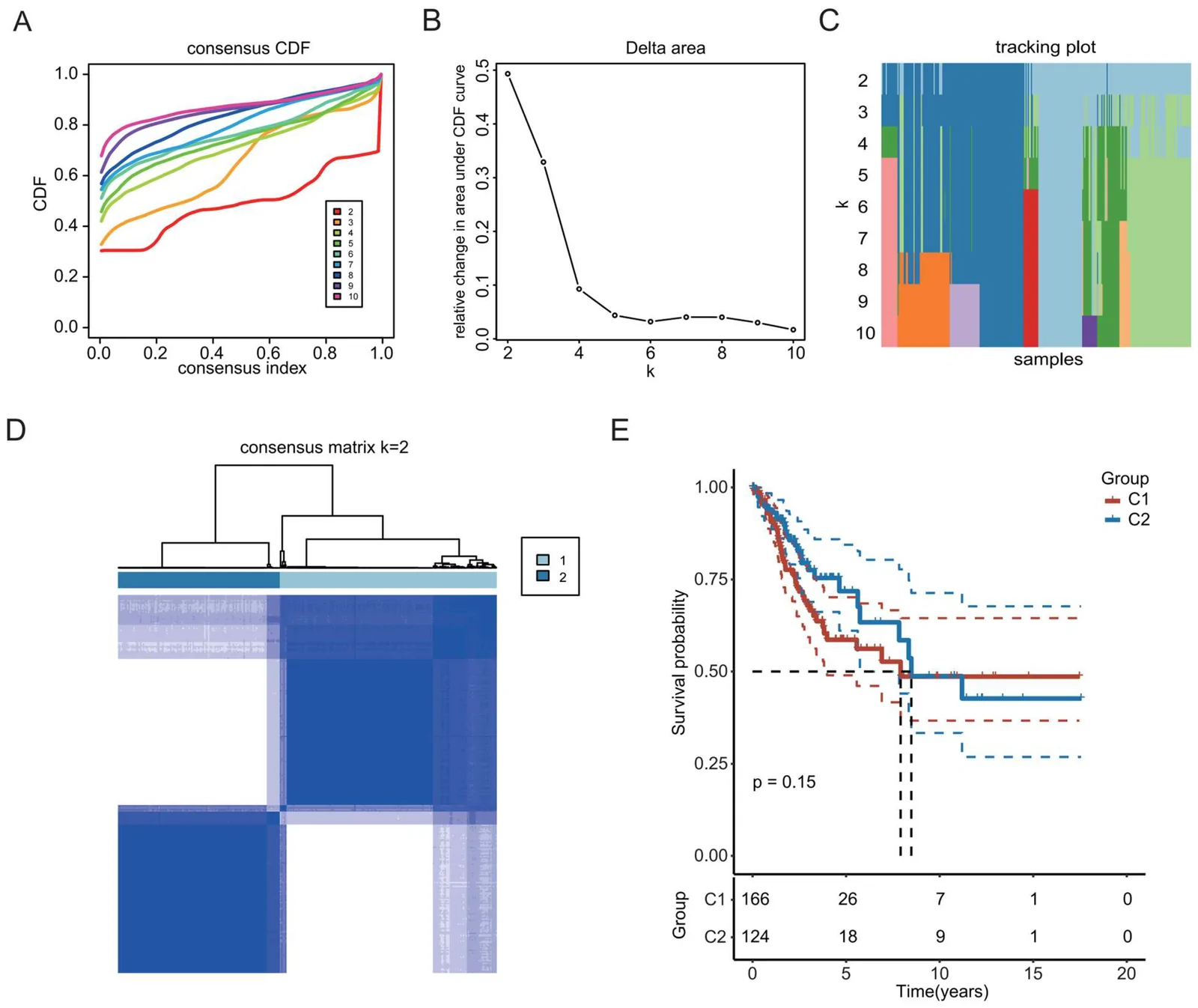
Two clusters based on consensus clustering of CRGs: (**A**) consensus CDF; (**B**) Delta area; (**C**) tracking plot; (**D**) consensus matrix when k = 2; (**E**) Kaplan–Meier survival curves for patients in the two clusters.

To characterize the expression patterns of CRGs in CC, patients were stratified into molecular subtypes based on consensus clustering of CRG expression. The consensus clustering analysis explored the relationship between cuproptosis and CC subtypes. Figure A2A illustrates the CDF (cumulative distribution function) for different numbers of clusters, K. Larger values indicate more robust clustering results for that value of K. Figure A2B shows the relative change in the area under the CDF curve compared to K and K − 1. According to the results, k = 2 showed the optimal clustering stability from k = 2 to 9 based on the similarity of expression (Figure A2C,D). The grouping case of the clustering is shown in Table S1. There was no statistically significant difference in the OS rate between cluster 1 and cluster 2 (*p* = 0.15) (Figure A2E), so we predicted the OS rate of patients using a new method—constructing a prognostic model by ML algorithms.

### 2.3. Machine Learning–Based Construction of the CRG Prognostic Models

**Figure 3:**
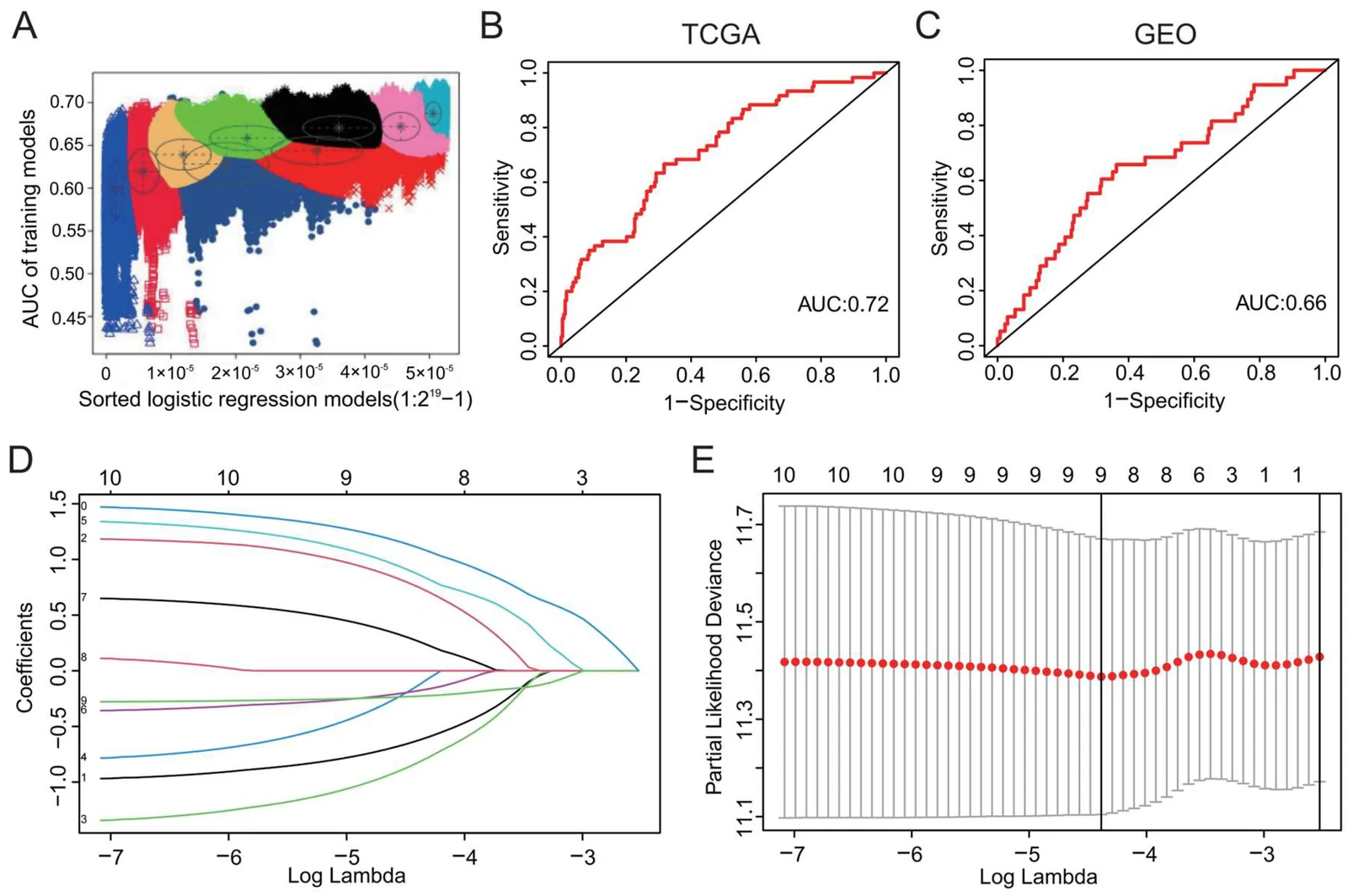
Identification of prognostic features and construction of risk scoring model: (**A**) GMM algorithm clustering. (**B**,**C**) The ROC curves of the GMM algorithm in the TCGA-CESC cohort (**B**) and GSE44001 cohort (**C**). (**D**) LASSO coefficient profiles of the 9 CRGs. Each curve corresponds to a gene. (**E**) Partial likelihood deviance for the LASSO coefficient profiles. The red dotted line stands for the cross-validation curve; error bars represent the upper and lower standard deviation curves along the λ sequence. The left vertical line shows the optimal λ value at which the minimum mean squared error is achieved and the corresponding genes. The right vertical line is for the most regularized model whose mean squared error is within 1 standard error of the minimum.

**Figure A3:**
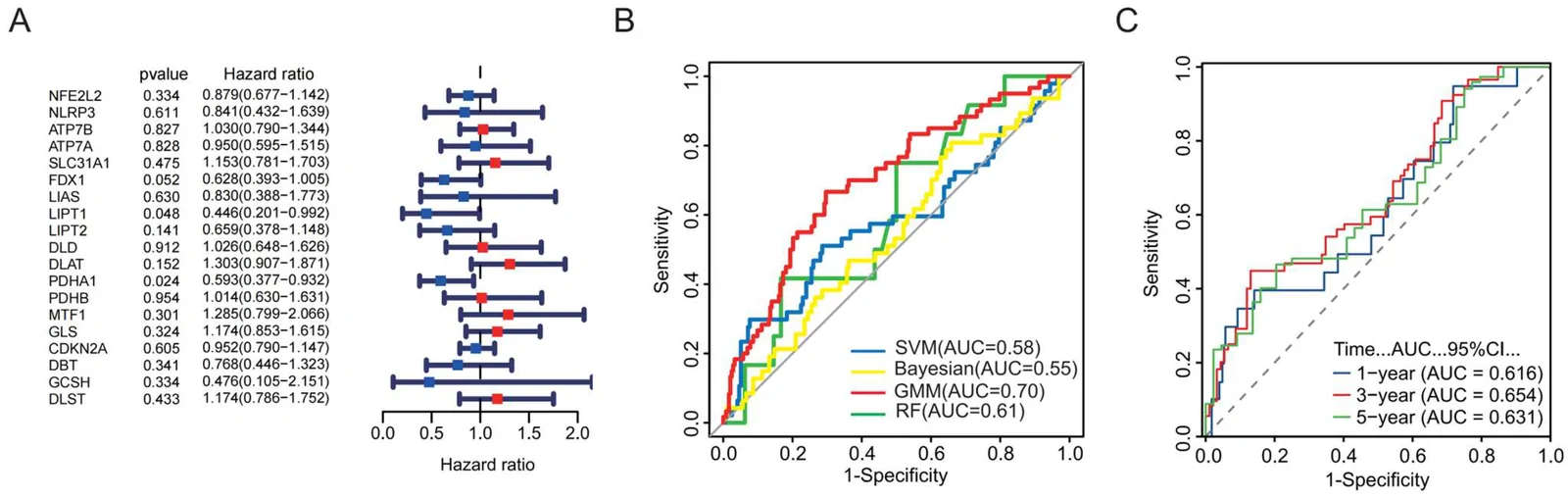
Accuracy of predictive risk model in CC: (**A**) the forest map indicated identified univariate Cox regression for 19 CRGs; (**B**) the predictive accuracy of the 11 CRGs which were selected by GMM algorithm were verified by the ROC curve; (**C**) the 1-, 3-, and 5-year ROC curves of the optimal model were 0.616, 0.654, and 0.631, respectively.

To enhance prognostic accuracy in patients with CC, we constructed prognostic risk score models. Univariate analysis of 19 CRGs showed that two genes (LIPT1 and PDHA1) were related to OS (*p* < 0.05); both were associated with a protective effect (HR < 1) (Figure A3A). The expression values of 19 CRGs in CC were input as feature values into five different ML algorithms (RF, SVM, GMM, Bayesian, and StepCox), and the results are shown in Table S2. Of those, the GMM algorithm demonstrated superior capacity in the training cohort (AUC = 0.723) compared with the SVM (AUC = 0.514), RF (AUC = 0.591), Bayesian (AUC = 0.580), and StepCox (AUC = 0.601) algorithms. We first permuted to construct the possible logistic regression models. Given 19 input features, a total of 2^19^ models were generated and clustered into nine modules (Figure 3A). When mclust = 3, the trained model has the maximum AUC value (AUC = 0.72) (Figure 3B). To determine the accuracy of the GMM algorithm, the AUC value was calculated for the validation cohort (AUC = 0.66) (Figure 3C); the performance in predicting survival was about the same. Therefore, the genes selected by the GMM algorithm will be used as candidates for the next construction of a prognostic risk model.

We further proved the superiority of the GMM algorithm by inputting the 11 genes screened as feature values into the other four algorithms once again. The results still show that the AUC value of the GMM algorithm is the largest (Figure A3B).

We performed LASSO–Cox analysis on 11 genes screened using the GMM algorithm to construct the prognostic risk model. According to Figure 3D,E, the vertical dashed line on the left represents the optimal value of the variable based on the minimum standardized value. In total, we selected nine genes to construct the prognostic model based on the optimal setting of the tuning parameter λ. The best cuproptosis-related CC prognostic risk score model was as follows: RS= −0.623359 × (expression of NFE2L2) + 0.7484913 × (expression of ATP7A) − 0.820472 × (expression of SLC31A1) − 0.126969 × (expression of LIAS) + 0.8624449 × (expression of LIPT1) − 0.178326 × (expression of DLAT) + 0.2620365 × (expression of PDHA1) − 0.228373 × (expression of CDKN2A) + 0.103211 × (expression of GCSH).

### 2.4. Evaluation of the Prognostic Risk Model and External Validation

**Figure 4:**
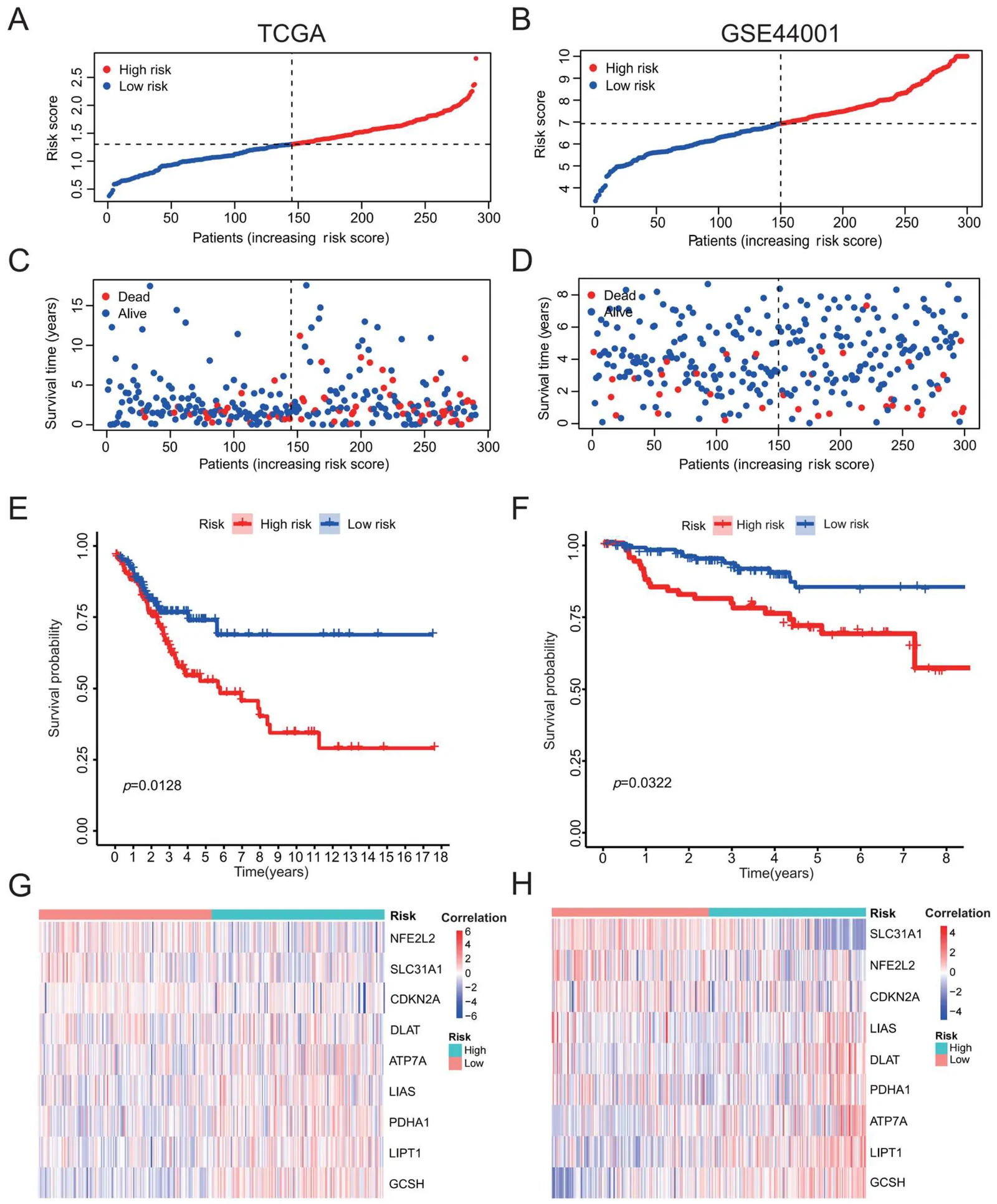
Predictive value of prognostic model in the training and test groups: (**A**,**B**) Risk curves of patients with different risk scores. (**C**,**D**) Scatterplots of patients with different survival statuses and survival times. (**E**,**F**) Kaplan–Meier survival curves of OS in different risk groups. (**G**,**H**) Heatmaps showing the expression of the 9 CRGs. Blue represents the low-risk group. Red represents the high-risk group.

A total of 291 patients with complete survival data were selected from the TCGA-CESC cohort as the training set for constructing the prognostic model. To demonstrate the reliability of the prognostic risk model, we divided the 291 patients into a high-risk group (*n* = 145) and a low-risk group (*n* = 146) according to the threshold of the median risk score in the training set (Figure 4A). The high-risk group had a higher mortality rate and shorter survival time compared with the low-risk group. The number of deaths increased with increasing risk score (Figure 4C). The KM curve depicted in Figure 4E indicates a favorable prognosis among patients exhibiting a lower RS. The time-dependent ROC analysis revealed that the prognostic accuracy for OS was 0.616 at 1 year, 0.654 at 3 years, and 0.631 at 5 years (Figure A3C). Figure 4G illustrates the expression of the nine characterized genes across the high-risk and low-risk patient groups, where GCSH, PDHA1, LIPT1, ATP7A, and LIAS are highly expressed in the high-risk group, and DLAT, CDKN2A, NFE2L2, and SLC31A1 are lowly expressed in the high-risk group.

To validate the accuracy of the prognostic model, we extracted 300 patients with CC from GSE44001 and calculated the RS using the same equation as the training set. On the basis of the median value of the RS, 150 patients were categorized into the low-risk group and 150 patients into the high-risk group (Figure 4B). Patients in the low-risk group had a longer survival time than those in the high-risk group (Figure 4D). Further, the KM analysis disclosed a significant discrepancy in survival rates between the low-risk and high-risk groups (*p* < 0.05, Figure 4F), which was in accordance with the result obtained in the training set. Consequently, we analyzed the model at a later stage.

### 2.5. Independent Prognostic Analysis of Risk Score and Clinical Characteristics

**Figure 5:**
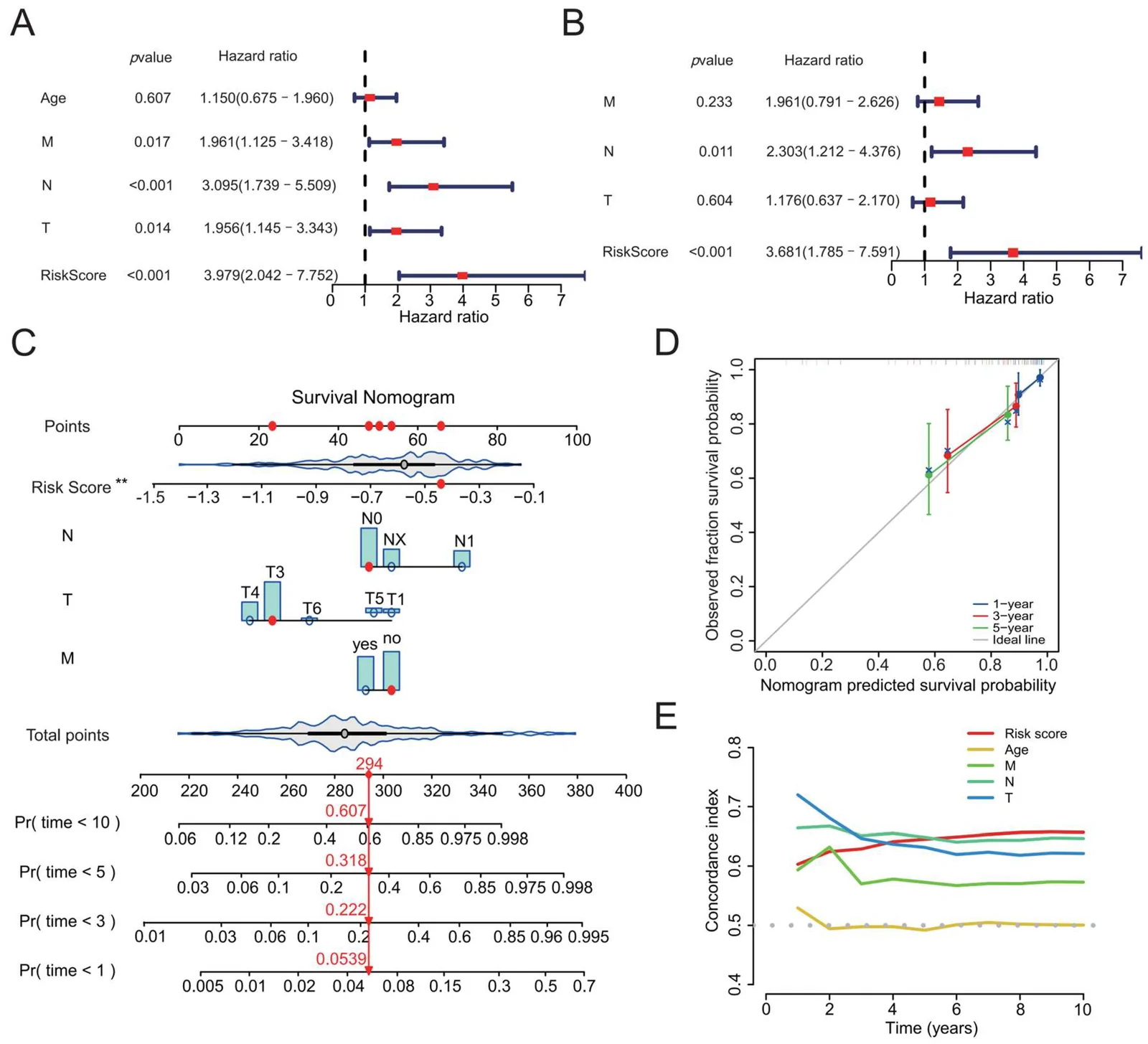
Construction and validation of nomogram for predicting survival probability in CC: (**A**,**B**) univariate and multivariate Cox regression analyses of the cuproptosis-related risk score and other clinical features to screen for factors independently associated with prognosis; (**C**) nomogram combining risk score with pathologic features; (**D**) calibration plots for predicting 1-, 3-, 5-year OS of patients; (**E**) a concordance index (C-index) was generated to assess the identification and forecasting capabilities of the nomogram. ** *p* < 0.01.

To verify whether the RS could serve as an independent prognostic factor, we performed univariate and multivariate analyses. Univariate and multivariate Cox regression analyses were performed on the RS and other clinical features, such as age and TNM stage. RS was independently correlated with OS in patients with CC, suggesting that it may act as an independent prognostic predictor with better prognostic value than clinical indicators (*p* < 0.001) (Figure 5A,B). Afterwards, a nomogram was constructed to derive a score for each factor based on patient-specific information and, ultimately, aiming to predict the patient’s OS probability based on the total score (Figure 5C). According to the nomogram, the RS was an exceedingly vital factor among various clinical parameters. We generated calibration curves to assess the accuracy of the nomogram in predicting the actual survival outcomes of patients with CC, demonstrating its high concordance with the actual survival of patients (Figure 5D). Furthermore, the prognostic risk model and TNM stage had a high differentiating capacity according to the concordance index (C-index) (Figure 5E).

### 2.6. Construction of the Cancer Staging Model for CC

**Figure 6:**
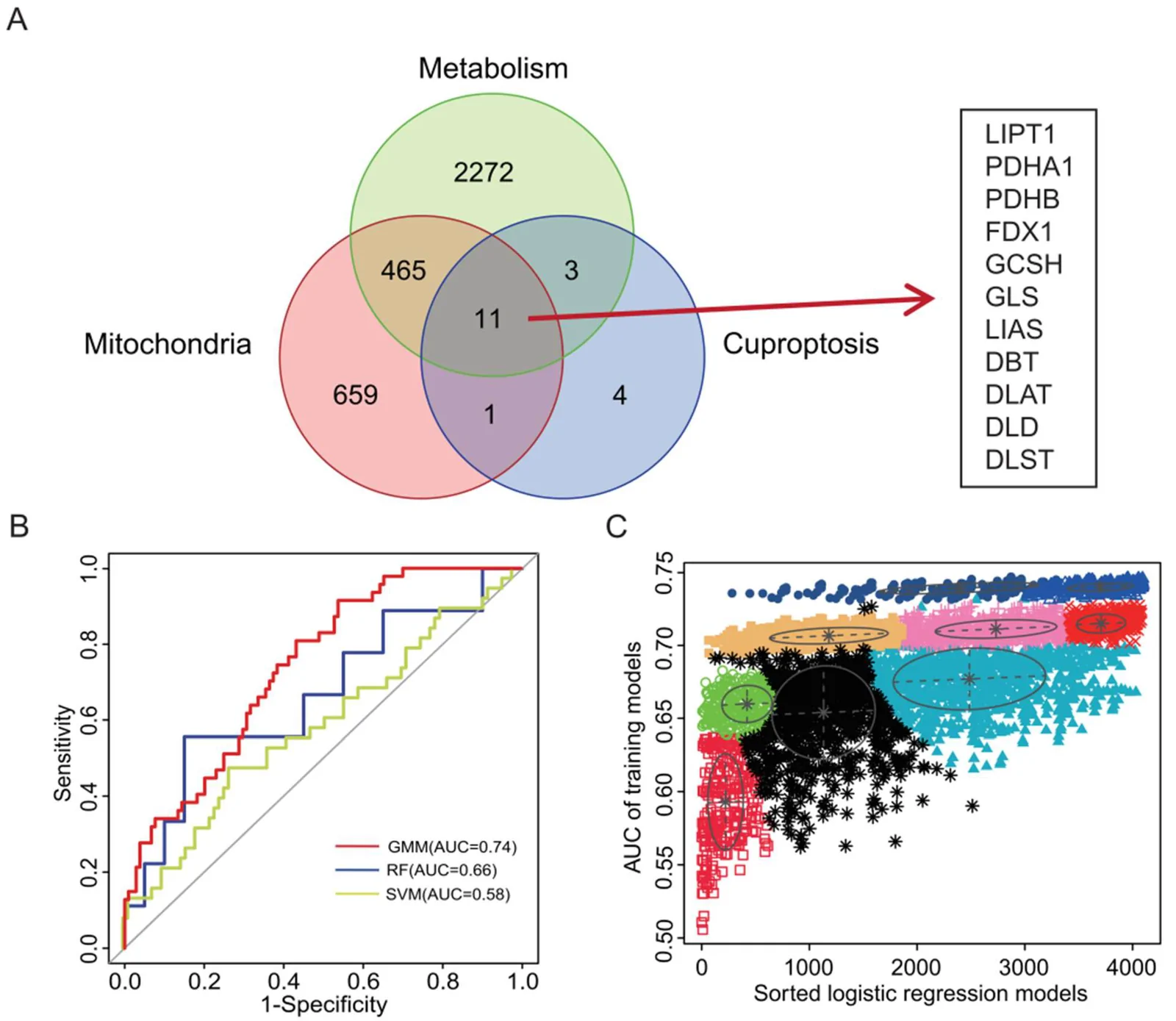
Construction of cuproptosis-related cancer staging model: (**A**) Venn diagram indicating the 11 genes identified in metabolism-related genes, mitochondria-related genes, and CRGs; (**B**) ROC analysis of the SVM, RF, and GMM algorithms, respectively; (**C**) GMM algorithm clustering.

**Figure A4:**
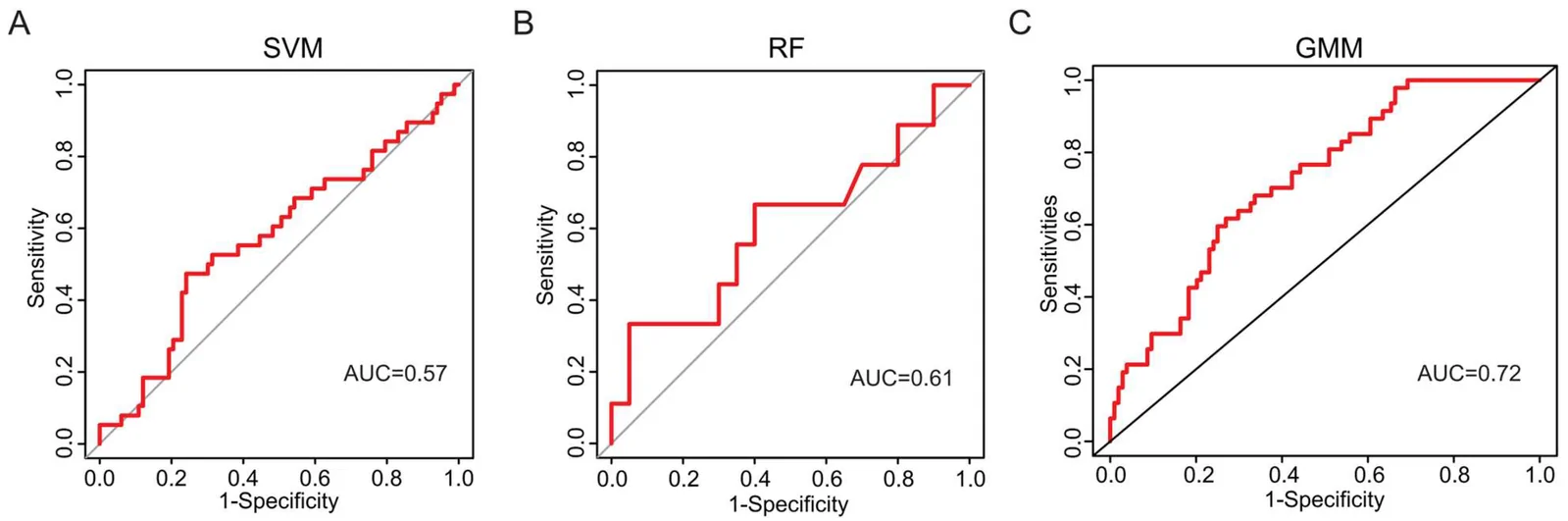
Accuracy of cancer staging model in CC: (**A**–**C**) ROC curves for prediction of 11-CRG cancer staging model by SVM (**A**), RF (**B**), and GMM (**C**).

Cancer staging is an important independent prognostic factor, so we constructed a cancer staging model associated with cuproptosis. Since the GO and KEGG analyses showed enrichment of the tricarboxylic acid cycle metabolic pathway and mitochondria were the site of execution (Figure 2E), we took 11 overlapping genes of CRGs, mitochondria-related genes, and metabolism-related genes, including LIPT1, PDHA1, PDHB, FDX1, GCSH, GLS, LIAS, DBT, DLAT, DLD, and DLST (Figure 6A). Subsequently, we used the expression of these 11 genes as feature values to input into each of the three ML algorithms (SVM, RF, and GMM), and their corresponding AUC values were 0.57, 0.61, and 0.72, respectively (Figure A4A–C). However, after many trials, we found an interesting observation: if we take these 11 genes together with CDKN2A as the input, then the AUC values of the three ML algorithms increase (SVM = 0.64, RF = 0.66, and GMM = 0.74) (Figure 6B). Therefore, these 12 genes would be used as characterization genes. By comparing the AUC values of the three ML algorithms, we constructed the cancer staging model using the GMM algorithm (Figure 6C).

### 2.7. Analysis of Functional Enrichment and TME

**Figure 7:**
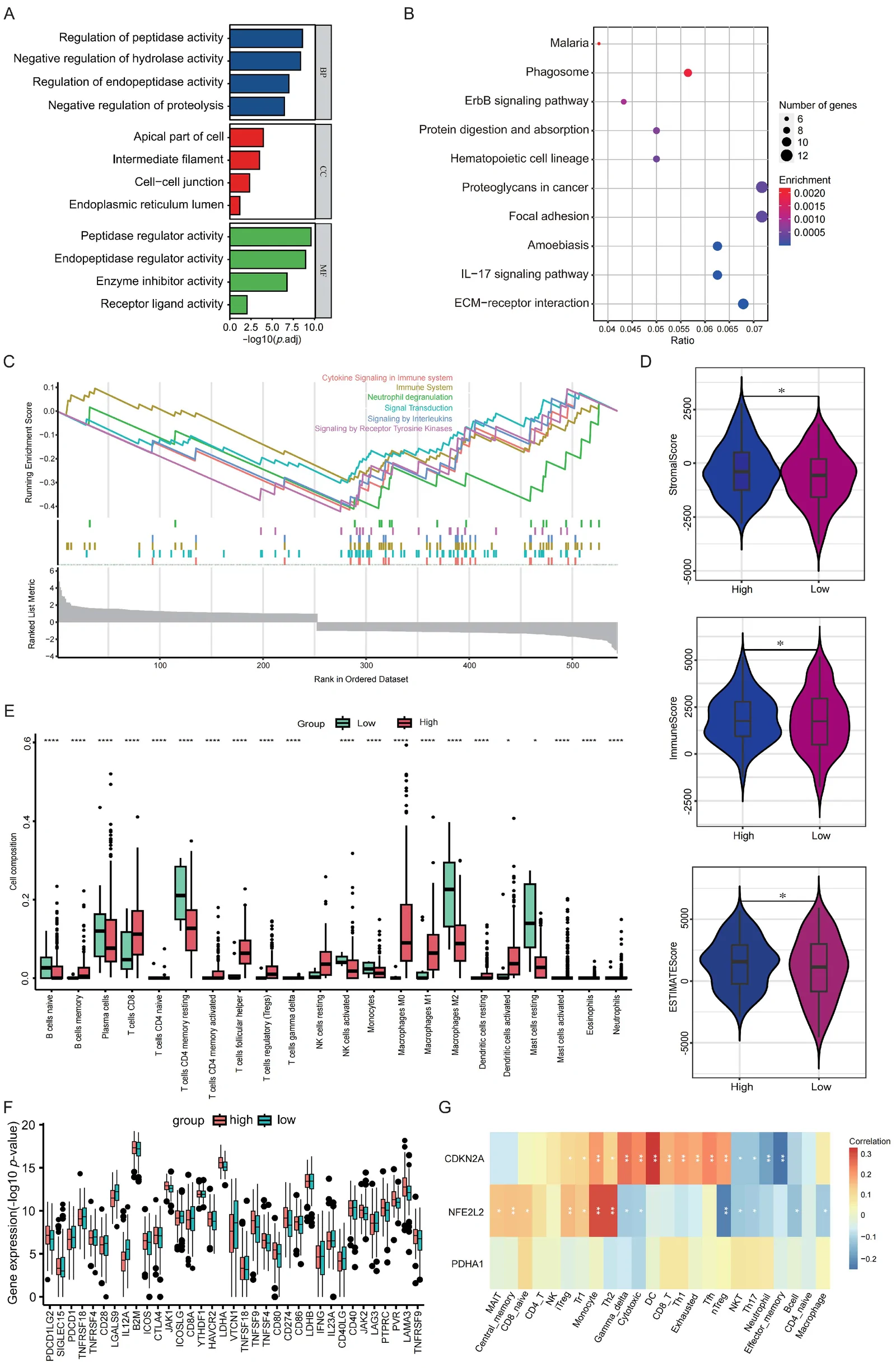
Functional annotation and analysis of TME and immune cell infiltration: (**A**) Most significant GO enrichment based on the differentially expressed genes between the two risk groups. (**B**) Results of KEGG pathway enrichment based on the differentially expressed genes between the two risk groups. (**C**) GSEA analysis functional enrichment results of differentially expressed genes in the high- and low-risk groups. (**D**) The relationships of risk score with stromal score, immune score, and ESTIMATE score. (**E**) Relative proportion of immune cell infiltration in high-risk and low-risk groups. Green represents the low-risk group. Red represents the high-risk group. (**F**) Comparison of the differences in expression of immune checkpoint–related genes between high- and low-risk groups. (**G**) Heatmap of the correlations between CDKN2A, PDHA1, NFE2L2, and immune checkpoint genes; * represents *p* < 0.05, ** represents *p* < 0.01, **** represents *p* < 0.0001.

We performed enrichment analysis of the differentially expressed genes between the high- and low-risk groups. The GO analysis results demonstrate that differentially expressed genes in the high- and low-risk groups are enriched in the negative regulation of proteolysis and the regulation of peptidase activity (Figure 7A). The KEGG analysis showed that the differentially expressed genes may be connected to the phagosome and IL-17 signaling pathways, suggesting that the genes were associated with immunity to tumors (Figure 7B). In addition, GSEA revealed that cytokine signaling in the immune system was enriched (Figure 7C).

Next, we performed immune cell infiltration analysis. We calculated immune score, stromal score, and ESTIMATE score to analyze the interactions between the scores and RS. The immune rejection score was higher in the high-risk group than in the low-risk group (Figure 7D). We discovered that M0 macrophages and NK cells were considerably enriched in patients with low risk (Figure 7E). Subsequently, by comparing the differences in expression of immune checkpoint–related genes between the high- and low-risk groups, researchers can gain further insights into the immune escape mechanisms of CC (Figure 7F). In Figure 7G, we present the relationships between the genes and the infiltration levels of 21 different immune cells in the form of a heatmap. Notably, there is a significant positive correlation between CDKN2A, PDHA1, NFE2L2, and NK cells, suggesting their potential roles in the immune microenvironment of CC.

### 2.8. Evaluating the Therapeutic Response in Patients

**Figure 8:**
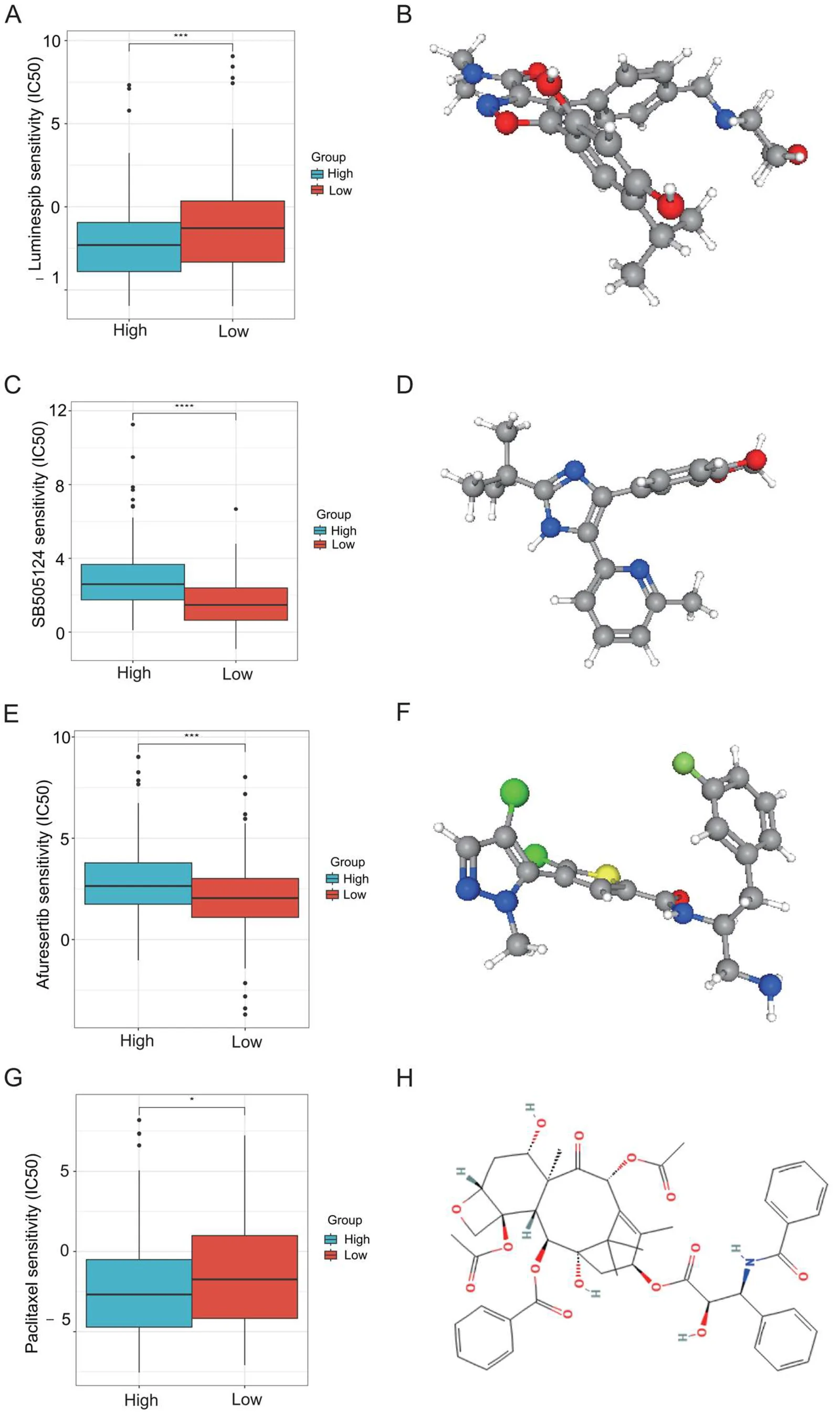
The screened drugs for CC treatment. IC50 values of Luminespib (**A**), SB505124 (**C**), Afuresertib (**E**), and Paclitaxel (**G**) in high- and low-risk patients with CC. The corresponding 3D structures are shown in (**B**,**D**,**F**), but (**H**) is two-dimensional conformation. * represents *p* < 0.05, *** represents *p* < 0.001, **** represents *p* < 0.0001.

We found that the half-maximal inhibitory concentration (IC50) values of Luminespib and Paclitaxel were lower in high-risk populations. Conversely, in low-risk populations, the IC50 values of SB505124 and Afuresertib showed noticeable reductions (Figure 8A,C,E,G). Figure 8B,D,F,H show the three-dimensional conformation of the four small-molecule compounds. Since the three-dimensional conformation of Paclitaxel was not found in the PubChem database, we showed its two-dimensional conformation.

### 2.9. Validation of the Expression of the Key CRG Signature in CC

**Figure 9:**
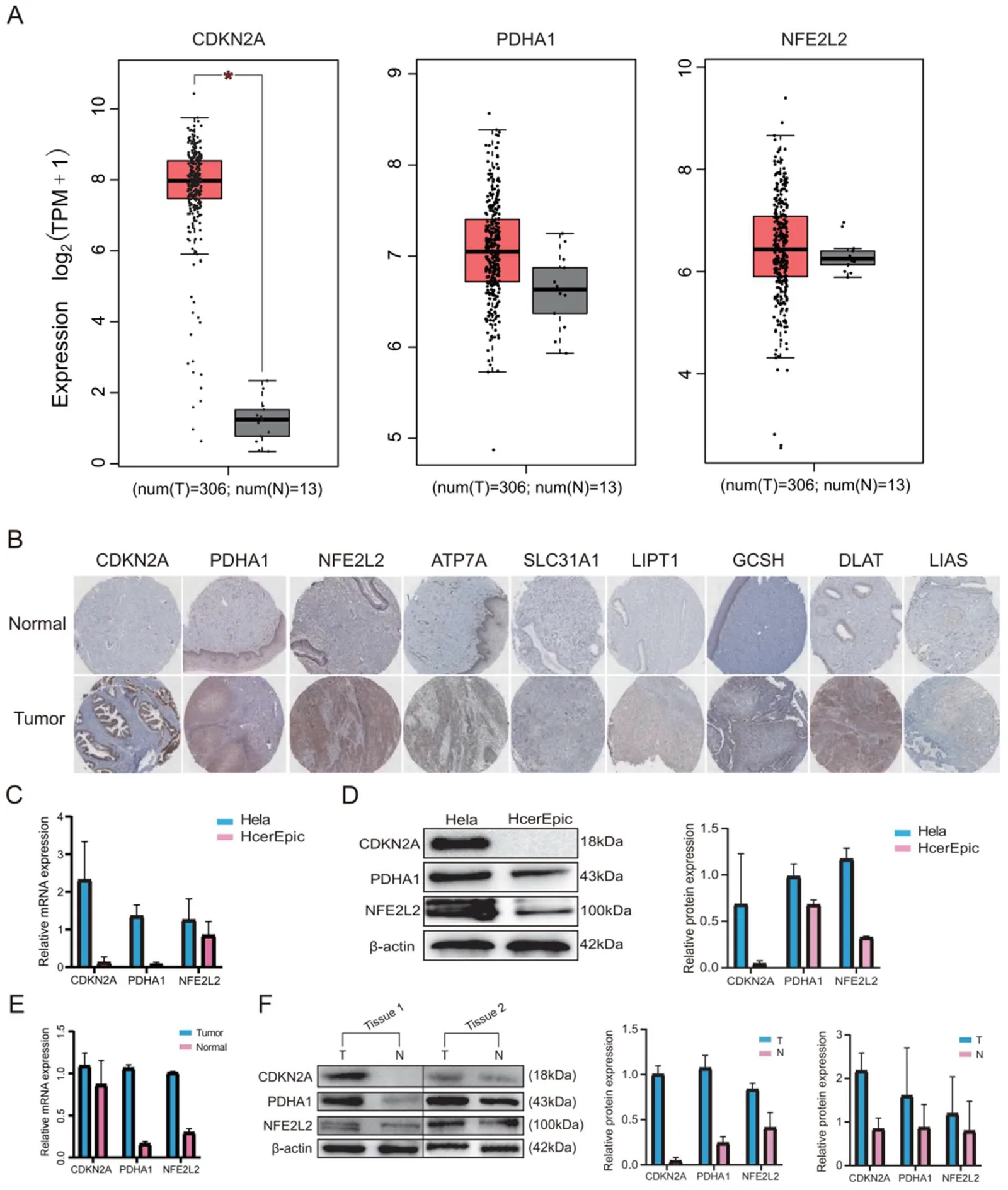
The relative expression of CRGs in CC and normal tissue samples: (**A**) differential expression of CDKN2A, NFE2L2, and PDHA1 genes between cervical cancer and normal tissues; (**B**) representative immunohistochemical staining for CDKN2A, PDHA1, NFE2L2, ATP7A, SLC31A1, LIPT1, GCSH, DLAT, and LIAS in normal and cervical tumor tissues; (**C**) the expression levels of CDKN2A, NFE2L2, and PDHA1 mRNAs were measured in CC cell lines and healthy cervical cell line; (**D**) Western blot results showing the expression levels of CDKN2A, NFE2L2, and PDHA1 in HeLa (**left panel**) or HcerEpic (**right panel**) cell lines, respectively; (**E**) the expression levels of CDKN2A, NFE2L2, and PDHA1 mRNAs were determined in tumor tissue and matched para-carcinoma tissue; (**F**) representative protein bands of CDKN2A, NFE2L2, and PDHA1 in cancer and para-carcinoma tissues. * represents *p* < 0.05.

CDKN2A demonstrates significant overexpression in CC relative to normal tissue, while PDHA1 and NFE2L2 exhibited a trend towards overexpression (Figure 9A). We retrieved immunohistochemical staining images of both normal cervical tissues and tumorous tissues from the Human Protein Atlas (HPA) database for further analysis. Figure 9B shows that the expression levels of CDKN2A, NFE2L2, and PDHA1 in the cervical tumor tissue are remarkably higher than those in the normal tissue. We further validated the expression of the key CRGs (CDKN2A, NFE2L2, and PDHA1) in CC cell lines and tissue. We first observed a significant decrease in the expression of CDKN2A, NFE2L2, and PDHA1 mRNA levels in HcerEpic cells in comparison with the levels in HeLa cells (Figure 9C). Western blot analysis further indicated that CDKN2A, NFE2L2, and PDHA1 protein levels were all higher in HeLa cells (Figure 9D). Furthermore, CDKN2A, NFE2L2, and PDHA1 proteins and mRNA levels were up-regulated in human CC tumor tissues in comparison with those in matched para-carcinoma tissues (Figure 9E,F).

### 2.10. The Inhibitory Effect of DDP on the Proliferation of Cervical Cancer Cells

**Figure 10:**
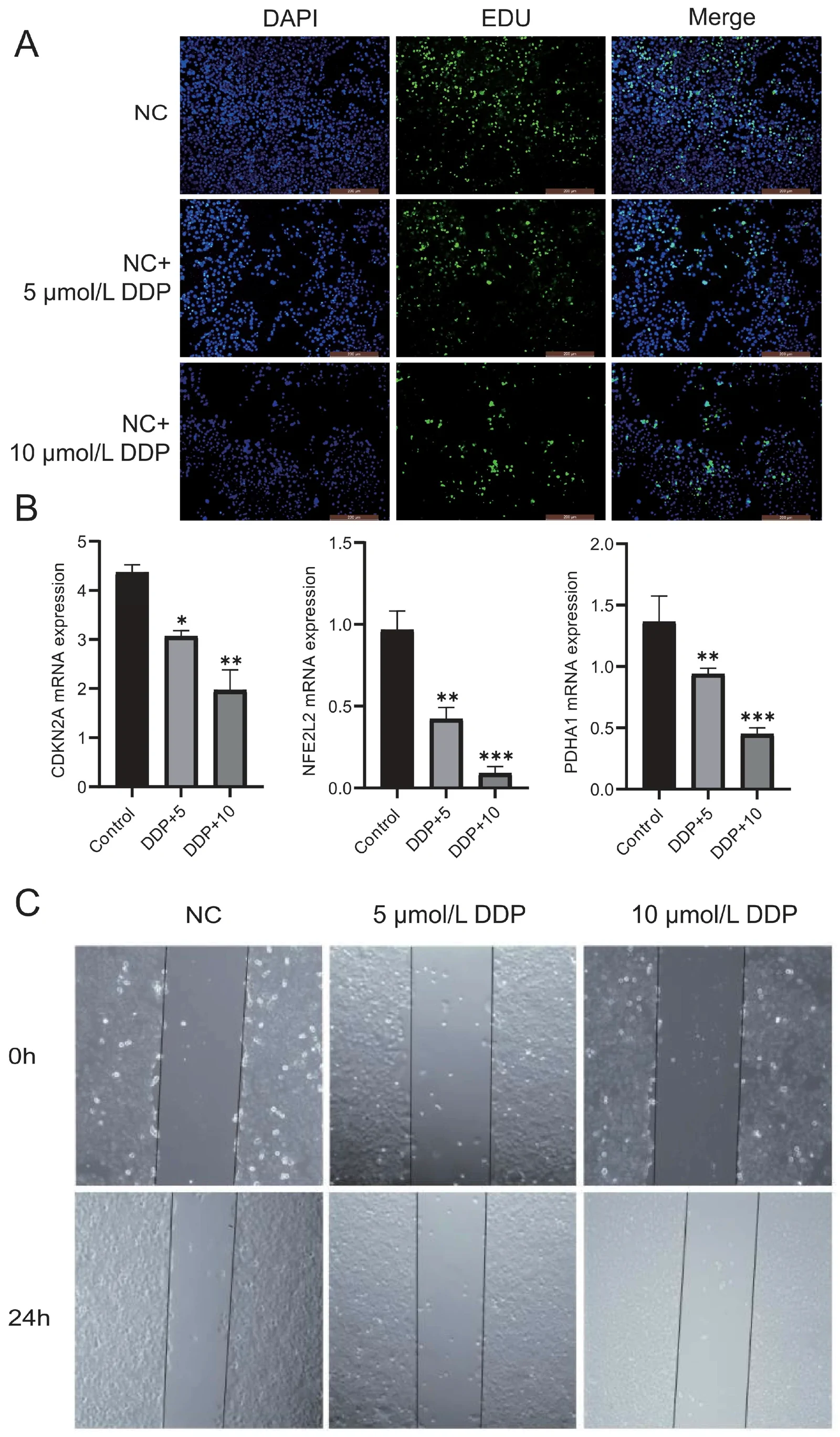
Cisplatin inhibits the proliferation of CC cells: (**A**) The EdU assay was performed to evaluate the effect of increasing DDP concentrations on CC cell proliferation rates; all scale bars are 200 μm. (**B**) Expression of CDKN2A, NFE2L2, and PDHA1 mRNAs after drug treatment. (**C**) The migration ability of HeLa cells in each group, observed for 24 h by wound healing assay. * represents *p* < 0.05, ** represents *p* < 0.01, *** represents *p* < 0.001.

To assess chemotherapeutic agent sensitivity between low-risk and high-risk populations, we screened candidate compounds and identified 42 small-molecule compounds showing significantly different sensitivity between the two groups (*p* < 0.05). Subsequently, the Comparative Toxicogenomics Database (CTD, https://ctdbase.org/, accessed on 23 August 2026) was searched for relationships between the 42 small-molecule compounds and CC. In total, 19 of them were more suitable for the treatment of CC, while drugs such as DDP, Paclitaxel, and Carboplatin are more appropriate for high-risk patient groups, as shown in Table S3. We examined the effects of DDP at different concentrations on the proliferation capacity of CC cells using the EdU assay. Compared with the control group, both low- and high-concentration DDP groups showed significantly reduced green fluorescence intensity. Moreover, the high-concentration DDP group exhibited a more pronounced decrease in fluorescence intensity than the low-concentration group (Figure 10A). The qRT-PCR results demonstrate that, compared with the control group, CC cells treated with DDP show significantly down-regulated mRNA expression of CDKN2A, NFE2L2, and PDHA1, and the downward trend was more obvious with the increase in DDP concentration (Figure 10B). DDP may inhibit the proliferation of CC by down-regulating the expression of CRGs. Wound healing assay results further indicated that the combination treatment was more effective in suppressing wound closure than either treatment alone (Figure 10C).

## 3. Discussion

Cuproptosis, triggered by the binding of copper to lipoylated proteins, represents a distinct form of regulated cell death mediated by a previously unrecognized molecular mechanism [30]. In recent years, it has become increasingly clear that cuproptosis plays an important role in cancer progression. Previous studies have found that the concentration of copper in cells affects a variety of cancers, such as breast cancer, head and neck cancer, and endometrial cancer [31]. Currently, not all patients with CC benefit from adequate treatments; for this reason, new biomarkers and methods are being developed to enable timely and effective therapies [32]. However, the specific roles of CRGs in CC remain incompletely understood. Accordingly, in this study, we established a prognostic risk score model based on CRGs. Feature genes were selected by RF, SVM, GMM, Bayesian, and StepCox algorithms. The GMM algorithm displayed better prognostic predictive performance, with an AUC of 0.72. A total of 11 cuproptosis-related feature genes (CDKN2A, LIPT1, NFE2L2, PDHA1, ATP7A, SLC31A1, LIAS, DLAT, GCSH, MTF1, and PDHB) were selected by the GMM algorithm and were used in LASSO–Cox regression to construct a novel cuproptosis-related prognostic model. A predictive nomogram was constructed by combining the prognostic risk model with clinicopathologic factors from the CC samples. The C-index and calibration curves demonstrated that the nomogram effectively predicts patient prognosis in CC. Therefore, CRGs can be used as potential prognostic biomarkers to provide new insights into potential therapeutic targets and therapeutic strategies for CC.

Cancer staging provides critical information regarding tumor location, size, and extent of invasion and significantly impacts overall survival (OS). Different treatments are used for different stages, which reduces medical stress and improves patient survival; therefore, we constructed a cancer staging model. Although the model was successfully developed, certain limitations persist, including an imbalanced sample size distribution, which may introduce unavoidable bias. In the future, collecting training sets with larger sample sizes and “0” and “1” equilibria will help to further improve our model.

Most existing prognostic models rely solely on the LASSO–Cox algorithm. In contrast, our study employed five ML algorithms to minimize the risk of overlooking important biomarkers. By comparing the AUC values of the five ML algorithms, the GMM model results were found to be optimal. Therefore, we used the feature genes selected by the GMM algorithm for the subsequent construction of the LASSO–Cox regression model. Our model demonstrated that ML algorithms have a wide range of applications in the clinic, improving prognostic accuracy and assisting clinicians in making more sound judgments. However, due to data limitations, key clinical covariates such as FIGO stage and HPV status were not integrated; external validation relied solely on the GEO dataset, lacking independent cohort validation from ICGC or institutional datasets. Future studies will develop more precise composite prognostic tools through clinico-molecular joint modeling and ML optimization, with independent validation using ICGC and institutional datasets to ensure generalizability.

LASSO–Cox analysis was performed, and CDKN2A, LIPT1, PDHA1, NFE2L2, ATP7A, SLC31A1, LIAS, GCSH, and DLAT were finally selected as feature genes to calculate the risk score. Among the differentially expressed genes between tumor and normal samples, CDKN2A shows aberrant expression that is closely linked to resistance to chemotherapeutic agents—including DDP and Paclitaxel—in CC. CDKN2A encodes the p16 protein, which plays a key role in cell differentiation, cellular senescence, and death [33]. As an oncogene [34], CDKN2A aberrant expression contributes to gynecological malignancies, such as uterine leiomyosarcoma and ovarian cancer [35,36]. Previous studies have shown that CDKN2A is highly methylated in cancers compared to healthy tissues. CDKN2A deficiency is strongly associated with poor prognosis in CC [37]. HPV-driven CC features E6/E7-mediated cell cycle dysregulation, leading to p16INK4a accumulation [38,39]. Encoded by CDKN2A, p16INK4a serves as a surrogate marker for HPV-related cervical tumorigenesis [40]. Upon copper ion accumulation triggering lipoylated protein aggregation, p16INK4A balances energy demands through mitochondrial metabolic reprogramming. PDHA1, with two α and two β subunits, is a tumor suppressor that produces acetyl-CoA in the tricarboxylic acid cycle by oxidizing pyruvate carboxylic acid [41]. NFE2L2 is also one of the most vital model genes. NFE2L2 expression is low in plasmacytoid, clear cell, and endometrioid ovarian cancers, but high in mucinous subtypes [42]. Hypoxia modulates copper transporter expression via HIF-1α, with NFE2L2 (Nrf2) serving as the primary antioxidant stress regulator, activated in the CC hypoxic microenvironment and thereby influencing cuproptosis sensitivity [43]. Furthermore, NFE2L2 activation promotes tumor immune evasion by modulating the expression of immune checkpoint molecules such as PD-L1. Subsequently, we selected two genes (CDKN2A and NFE2L2) with high expression for validation in both tumor and normal cells. The results showed that they were highly expressed in CC cells, which was consistent with the results of our bioinformatics analyses. Our findings indicate that DDP exerts a dose-dependent inhibitory effect on CC cell proliferation and migration, accompanied by down-regulation of CDKN2A, NFE2L2, and PDHA1 expression, suggesting that CRGs may partially mediate the chemosensitivity of CC cells to DDP.

Studies show that clinical models of tumor-specific and immune microenvironments are crucial for understanding cancer pathogenesis, disease development, and response to therapy [44]. The results showed that the immune rejection score in the high-risk group was larger than in the low-risk group. Therefore, patients with CC in the low-risk group may receive immunotherapy with worse efficacy, while patients in the high-risk group are more suitable for receiving immunotherapy. Recent advantages suggest that immune checkpoint inhibitors might represent the new standard of care in patients with CC [45]. Interestingly, the addition of antibody–drug conjugates (ADCs) to chemotherapy and immunotherapy might improve the outcomes of those patients. Tumor-infiltrating lymphocytes (TILs) are emerging therapies tested for different solid tumors, such as CC [46].

At the same time, we screened out three potential small-molecule compounds, including Luminespib, SB505124, and Afuresertib. Luminespib is a second-generation heat shock protein 90 (HSP90) inhibitor [47] that can decrease the protein expression of membrane receptors, such as EGFR, MET, and AXL [48]. Studies have shown that, in liver carcinoma cells, Luminespib induces apoptosis via caspase activation and β-catenin fragmentation in HepG2 cells, while also suppressing tumor growth in vivo, positioning it as a promising therapeutic agent for CC [49]. The selective ALK5 inhibitor SB505124 acts as an ABE activator [50]. SB505124 down-regulated the TGF-β signaling pathway by decreasing phosphorylation of Smad2 [51]. Afuresertib is closely correlated with the AKT pathway. Therefore, it has been demonstrated that these drugs may provide new insights into the treatment of patients with CC.

In summary, our study establishes two robust CRG-based models for predicting CC prognosis and staging, with the GMM algorithm demonstrating superior performance. DDP dose-dependently suppressed CC proliferation and down-regulated CDKN2A, NFE2L2, and PDHA1, suggesting that CRGs may mediate chemosensitivity. However, its limitations, including retrospective data, a limited sample size, unbalanced FIGO distribution, and a lack of independent external validation, should be noted. Future prospective multicenter studies and mechanistic investigations are warranted to validate clinical utility and explore cuproptosis-targeted therapeutic strategies.

## 4. Materials and Methods

### 4.1. Data Acquisition

RNA-seq FPKM data of 304 patients with CESC (cervical squamous cell carcinoma and endocervical adenocarcinoma) and 3 normal samples were downloaded from The Cancer Genome Atlas (TCGA, https://portal.gdc.cancer.gov/, accessed on 3 March 2025). Additionally, 300 CC samples were downloaded from the Gene Expression Omnibus (GEO, http://www.ncbi.nlm.nih.gov/geo/, accessed on 5 March 2025) database (GSE44001). We also acquired the data of 10 normal tissue samples from Genotype–Tissue Expression (GTEx, https://portal.gdc.cancer.gov/, accessed on 3 March 2025). We collected clinical information on patients with CC, including age, grade, tumor–node–metastasis stage, survival status, and follow-up time (Table S4). At the same time, we collected FIGO staging for the GSE38964 dataset (GPL6884, *n* = 151). Based on the cuproptosis process described in the prior study [20,52], a total of 19 CRGs (FDX1, LIAS, LIPT1, LIPT2, GCSH, GLS, DBT, DLST, DLD, DLAT, PDHA1, PDHB, SLC31A1, ATP7A, ATP7B, NFE2L2, NLRP3, MTF1, and CDKN2A) were selected for further analysis. A total of 1136 mitochondrial-related genes were extracted from the MitoCarta 3.0 database (https://www.gsea-msigdb.org/gsea/msigdb (accessed on 15 March 2025)). Metabolism-related genes (*n* = 2752) were compiled from the previous literature and are detailed in Table S5.

### 4.2. Data Preprocessing

For integrating the expression data from the TCGA and GEO platforms, we performed batch normalization using the ComBat function in the “sva” R package to eliminate batch effects. Prior to batch correction, low-expression genes were filtered out based on the following criterion: genes with normalized count values < 1 in more than 50% of all samples were excluded from further analysis. This ensured that only reliably expressed genes were retained for downstream analyses. After excluding samples with missing prognosis information or invalid (zero) survival data, 291 samples from TCGA and 300 samples from GSE44001 were ultimately included in the follow-up study. The GSE38964 dataset was processed in the same manner, yielding a total of 151 samples for constructing the cancer staging model, among which FIGO stages I and II were classified as early-stage CC (*n* = 104), and stages III and IV as advanced-stage CC (*n* = 47).

### 4.3. Machine Learning Extraction of Significant Genes

The expression matrix generated from the expression of 19 CRGs was used as an input for screening the significant factors using five ML algorithms, including RF, SVM, GMM, Bayesian, and StepCox. Each of the five algorithms used the “randomForest” (v4.7-1.1), “e1071” (v1.7-14), “mclust” (6.0.1), “naivebayes” (v0.9.7), and “StepReg” (v.1.6.0) packages of the R language (v4.4.2). A fixed random seed of 12345 was used to improve the reproducibility of all results. We tested the accuracy of the algorithm using five-fold cross-validation. In the GMM algorithm, the “SimDesign” (v2.12.0) package was employed to reduce random variation in the results. Significant features identified by each algorithm were compared based on area under the curve (AUC) values to determine optimal feature selection.

### 4.4. Construction and Validation of the Prognostic Risk Score Model

The significant factors were used to construct a risk model by using the LASSO regression algorithm, which was implemented using the “glmnet” (4.1-8) R package. The weighting of the standardized expression values of the individual risk genes with the corresponding regression coefficients resulted in a risk score (RS) for each patient. The equation RS = Σ Coefi × Xi (where Coefi is the regression coefficient and Xi represents the gene expression value) was utilized. The GEO dataset was employed for validation. Given that the median cutoff is objective, simple, widely applicable in survival analyses, and ensures balanced sample sizes between groups, the patients with CC were divided into low-risk and high-risk groups based on the median RS. Using the “survival” R package, Kaplan–Meier (KM) curves were constructed to estimate OS in the two risk subgroups, while the accuracy of the risk score model was tested using the ROC curve.

### 4.5. Independent Prognostic Analysis

Univariate and multivariate Cox regression analyses were performed to identify independent prognostic predictors from the TCGA dataset, incorporating risk score and clinical characteristics. A prognostic nomogram was subsequently constructed to predict patient prognosis. The predictive accuracy of the nomogram was assessed using calibration curves and concordance indices.

### 4.6. Construction of the Cancer Staging Model

To screen the optimal eigenvalues for constructing the cancer staging model, 19 CRGs were intersected with the 2752 metabolism-related genes and 1136 mitochondria-related genes, and these genes served as characteristic genes. The cancer staging model was constructed using three ML algorithms, including SVM, RF, and GMM. Gene expression values from GSE38964 were used as input features, with FIGO stages I and II coded as “0” and stages III and IV coded as “1”. The GSE38964 cohort was randomly divided into a training cohort and test cohort at a 4:1 ratio.

### 4.7. Function Enrichment Analysis

Differential expression analysis between the tumor and normal tissues was performed using the “limma” R package. Statistically significant differentially expressed genes were determined based on an adjusted *p*-value < 0.05 and |log2FC| ≥ 1. Gene Ontology (GO) and Kyoto Encyclopedia of Genes and Genomes (KEGG) pathway enrichment analyses were conducted on the CRGs, including the top 40 differentially expressed genes (20 up-regulated and 20 down-regulated). Gene Set Enrichment Analysis (GSEA) was applied to identify significantly enriched pathways and functional categories associated with the risk models [53].

### 4.8. Immune Cell Infiltration Analysis

To validate variations in microenvironment features across distinct risk subgroups, the “estimate” R package was utilized to conduct an analysis of the immunological and stromal components within the TME of each CC sample. Utilizing the “CIBERSORT” R package, we extracted and quantified the relative percentages of 22 distinct types of infiltrating immune cells present in each CC sample. Furthermore, we examined the correlation between the genes identified by ML and the immune checkpoint genes.

### 4.9. Drug Sensitivity Analysis

To evaluate the sensitivity of individual patients with CC to chemotherapeutic agents, we utilized the Genomics of Drug Sensitivity in Cancer (GDSC, https://www.cancerrxgene.org/, accessed on 23 August 2026) database, and the IC50 was used for quantification using the “oncoPredict” package of R. Then, we used PubChem (https://pubchem.ncbi.nlm.nih.gov/, accessed on 23 August 2026) to visualize the conformations of drugs in 3D. We calculated drug and cervical inference scores from the Comparative Toxicogenomics Database (CTD, https://ctdbase.org, accessed on 23 August 2026).

### 4.10. The Human Protein Atlas Analysis

The Human Protein Atlas (HPA, https://www.proteinatlas.org/, accessed on 23 August 2026) database provides a wealth of information on the expression, localization, and function of human proteins in a variety of tissues and cell types [54]. In the study, we obtained the pathological tissue staining map of the gene signature in CC from the HPA database and observed whether these markers differ between normal and tumor tissues.

### 4.11. Collection of Tissue Specimens

We obtained cancer tissues and matched para-carcinoma tissues from 3 patients with CC who underwent surgical treatment at the First Affiliated Hospital of Jiamusi University. All tissue specimens were freshly collected and immediately snap-frozen in liquid nitrogen after resection, and subsequently stored at −80 °C until further use. None of the patients received radiotherapy/chemotherapy or other anti-tumor treatment prior to surgery. All experiments in this study were approved by the Ethics Committee of the First Affiliated Hospital of Jiamusi University (ethical approval number: 202411).

### 4.12. Cell Culture

The human cervical epithelial cell line (HCerEpiC) was acquired from the American Type Culture Collection (ATCC), while CC cell lines, including HeLa, were obtained from the Cell Bank of the Chinese Academy of Medical Sciences. HCerEpiC cells were cultured in Dulbecco’s Modified Eagle Medium (DMEM), enriched with 15% FBS, 1% penicillin–streptomycin, and 1% HEPES buffer. The cultures were maintained in a constant-temperature incubator at 37 °C, 5% CO_2_, and optimal humidity. The medium was refreshed every two days, and cells were passaged when reaching 80% confluence. The experiments were conducted using cells in the logarithmic growth phase.

### 4.13. EdU Assay

DNA synthesis rate assessment was conducted utilizing Click-iT EdU Imaging Kits (Beyotime, Beijing, China), and experiments were performed using the method provided by the manufacturer. The HeLa cell line was treated with different concentrations of DDP (0, 5, and 10 μmol/L) for 24 h, and incubated with EdU (20 mmol/L) for 2 h. The cells were fixed with 4% paraformaldehyde for 20 min at room temperature. EdU-positive cells were analyzed across different treatment groups.

### 4.14. RNA Extraction and Quantitative Real-Time PCR (qPCR)

Cellular total RNA was isolated using Trizol (Invitrogen, Carlsbad, CA, USA). Total RNA was reverse-transcribed into cDNA. Relative RNA expression was detected by SYBR Green PCR (Zoman Biotechnology, Beijing, China). The primers used in the real-time qPCR assays are listed in Table S6. All qRT-PCR experiments were performed with three biological replicates and three technical replicates for each sample. Data are presented as mean ± standard deviation (SD). Statistical comparisons between two groups were performed using a two-tailed Student’s *t*-test, with *p* < 0.05 considered statistically significant.

### 4.15. Western Blot Analysis

Tissue specimens or cell lines were used to extract total protein samples with RIPA lysis buffer (RIPA:PMSF = 100:1) with a cocktail of inhibitors. The cell lysate was centrifuged at 12,000 rpm for 15 min at low temperature, and the supernatant was collected. The protein concentration was measured using the BCA method. The proteins were separated by 12% SDS-polyacrylamide gel electrophoresis and transferred to a PVDF membrane. After blocking with 5% skimmed milk on a shaker for 1 h, the primary antibodies β-actin (1:2000), NFE2L2 (1:5000), CDKN2A (1:2000), and PDHA1 (1:5000) were added separately and incubated overnight at 4 °C. The membrane was washed with TBST for 10 min, 3 times, and then HRP-conjugated secondary antibodies (1:1500) were added and incubated at room temperature for 1 h. The membrane was washed with TBST for 10 min, 3 times. ECL luminescent liquid was used for visualization in a gel imaging system. All Western blot experiments were independently repeated three times using protein lysates prepared from three independent biological replicates. Data are presented as mean ± SD. Statistical comparisons between two groups were performed using a two-tailed Student’s *t*-test, with *p* < 0.05 considered statistically significant.

## 5. Conclusions

In conclusion, this study not only constructed two models based on CRGs for the prognosis and cancer staging, but also reinforces the importance of cuproptosis in CC. We constructed, for the first time, a CRG-based cancer staging model using the GMM algorithm, which accurately predicts the stage of CC. The results demonstrate some value, and a nomogram of this prognostic model may be useful in predicting the risk of disease progression in individual patients. Furthermore, CDKN2A, NFE2L2, and PDHA1 are significantly overexpressed in CC, and DDP suppresses CC cell proliferation—an effect potentially mediated via CRG regulation. Collectively, these findings highlight the clinical relevance of CRG-based models and identify cuproptosis as a promising therapeutic target in CC. The ML-based analytical strategy described herein warrants broader application in precision medicine. Although significant progress has been made in the treatment of patients with CC in recent years, the heterogeneity and complexity of the disease necessitate further research into new drugs and the development of personalized treatment plans tailored to individual patients in the future.

## Data Availability

Data for TCGA-STAD patients and normal samples were downloaded from The Cancer Genome Atlas (TCGA, https://portal.gdc.cancer.gov/, accessed on 3 March 2025). GC sample data were retrieved from the GSE38964 and GSE44001 datasets in the Gene Expression Omnibus (GEO) database (http://www.ncbi.nlm.nih.gov/geo/, accessed on 5 March 2025). Normal tissue data were obtained from the Genotype–Tissue Expression (GTEx) project (https://gtexportal.org/home/, accessed on 23 August 2026).

## Supplementary Materials

The following supporting information can be downloaded at: https://www.mdpi.com/article/10.3390/ijms27177730/s1.

## Abbreviations

The following abbreviations are used in this manuscript:

CC	Cervical cancer
CRGs	Cuproptosis-related genes
ML	Machine learning
RF	Random forest
SVM	Support vector machine
GMM	Gaussian mixture model
OS	Overall survival
HPV	Human papillomavirus
AI	Artificial intelligence
CTD	Comparative Toxicogenomics Database
C-index	Concordance index
GEO	Gene Expression Omnibus
TME	Tumor microenvironment
DDP	Cisplatin
ADCs	Antibody–drug conjugates
CESC	Cervical squamous cell carcinoma and endocervical adenocarcinoma
GTEx	Genotype–Tissue Expression
KM	Kaplan–Meier
KEGG	Kyoto Encyclopedia of Genes and Genomes
GO	Gene Ontology
GSEA	Gene Set Enrichment Analysis
